# SENP1 regulates PTEN stability to dictate prostate cancer development

**DOI:** 10.18632/oncotarget.13283

**Published:** 2016-11-10

**Authors:** Tasneem Bawa-Khalfe, Feng-Ming Yang, Joan Ritho, Hui-Kuan Lin, Jinke Cheng, Edward T.H. Yeh

**Affiliations:** ^1^ Department of Biology & Biochemistry, Center for Nuclear Receptors & Cell Signaling, University of Houston, Houston, Texas, USA; ^2^ Department of Internal Medicine, The University of Missouri, Columbia, MO, USA; ^3^ Department of Molecular and Cellular Oncology, The University of Texas MD Anderson Cancer Center, Houston, Texas, USA; ^4^ Department of Cancer Biology, Wake Forest School of Medicine, Winston-Salem, North Carolina, USA; ^5^ Department of Biochemistry and Molecular Cell Biology, Key Laboratory of Cell Differentiation and Apoptosis of Chinese Ministry of Education, Shanghai Jiao Tong University School of Medicine, Shanghai, China; ^6^ State Key Laboratory of Oncogenes & Related Genes, Shanghai Cancer Institute, Shanghai Jiao Tong University School of Medicine, Shanghai, China

**Keywords:** SENP1, PTEN, WWP2, SUMO, prostate carcinogenesis

## Abstract

SUMO protease SENP1 is elevated in multiple carcinomas including prostate cancer (PCa). SENP1 exhibits carcinogenic properties; it promotes androgen receptor-dependent and -independent cell proliferation, stabilizes HIF1a, increases VEGF, and supports angiogenesis. However, mice expressing an androgen-responsive promoter driven SENP1-transgene (SENP1-Tg) develop high-grade prostatic intraepithelial neoplasia, but not carcinoma. We now show that tumor suppressive PTEN signaling is induced in SENP1-Tg to enhance prostate epithelial cell apoptosis. SENP1 blocks SUMO1-dependent ubiquitylation and degradation of PTEN. In the absence of SENP1, SUMO1-modified PTEN is sequestered in the cytosol, where binding to ubiquitin-E3 ligase WWP2 occurs. Concurrently, WWP2 is also SUMOylated, which potentiates its interaction with PTEN. Thus, SENP1 directs ubiquitin-E3-substrate association to control PTEN stability. PTEN serves as a barrier for SENP1-mediated prostate carcinogenesis as SENP1-Tg mice develop invasive carcinomas only after PTEN reduction. Hence, SENP1 modulates multiple facets of carcinogenesis and may serve as a target specifically for aggressive PTEN-deficient PCa.

## INTRODUCTION

SUMO (small-ubiquitin-like modifier) posttranslational modification (PTM) occurs on numerous substrates to affect the protein's function, subcellular localization, and/or stability. Hence SUMO conjugation or SUMOylation is an important regulator of normal/physiological and pathophysiological cellular events. This PTM is dynamic and the normal kinetics is closely guarded *via* the activity of SUMO-specific conjugating and deconjugating enzymes. Imbalance in the level of SUMOylated substrates often occurs with and supports the cancer microenvironment [1]. Hence, components of the SUMO machinery present attractive subjects for targeted-cancer therapy.

We and others report transcriptional induction of the Sentrin/SUMO-specific isopeptidase SENP1 in human pre-cancer lesions or prostatic intraepithelial neoplasia (PIN) and correlative elevation of SENP1 protein with enhanced prostate cancer (PCa) malignancy [2–5]. In human PCa cells, SENP1 controls critical mediators of PCa formation and progression specifically the androgen receptor, hypoxia-inducible factor 1a (HIF1α), c-jun, and cyclin D1 [3, 5–7]. These observations caused the recent drive to develop SENP1-selective inhibitors [8, 9]. However, engineered mice with selective expression of the SENP1 transgene in the epithelia of the prostate gland (PG) do not develop cancer [2]. The SENP1-transgenic mice (SENP1-Tg) do exhibit aberrant transformation of the mouse PG indicative of high-grade PIN. The transformed PG of the SENP1-Tg mouse exhibits induction of the same pro-oncogenic and -angiogenic pathways identified in PCa cell models and named above. Since SENP1 can and does regulate multiple mechanisms simultaneously, it is highly probable that the absence of carcinoma in the SENP1-Tg mouse model is due to a concurrent SENP1-regulation of unidentified tumor-suppressive pathway(s).

PTEN (phosphatase and tensin homologue deleted on chromosome ten) is a well-established tumor suppressor that has phosphatase activity against lipids and proteins. Although initially defined as a cytosolic protein, recent studies have shown that PTEN exhibits nucleocytosolic trafficking. Nuclear PTEN initiates multiple pathways to support cell apoptosis and therefore, is primarily responsible for its anti-tumor effects [10]. Consistently, expression of cytosolic PTEN, the less efficient apoptotic mediator, correlates with highly aggressive carcinomas [11–13]. In the PCa epithelia, *PTEN* mutation and loss of heterozygosity significantly reduce PTEN protein levels and thereby negate the tumor suppressive properties of nuclear PTEN.

Ectopic PTEN in PCa cells is subject to multiple PTMs including ubiquitylation and more recently, SUMOylation. Interestingly, both ubiquitin and SUMO modifications direct PTEN's subcellular localization [10, 14–16]. Cytosolic PTEN is poly-ubiquitylated and targeted for proteasomal degradation while mono-ubiquitylation causes translocation of PTEN to the nucleus [16]; what promotes poly- over mono-ubiquitylation of PTEN is unknown. It is known that the NEDD4 family of ubiquitin E3 ligases, specifically NEDD4-1 and WWP2, mediate PTEN ubiquitylation. In PCa cells, WWP2 is the major E3 ligase for ubiquitin-mediated PTEN degradation [17, 18]. Additionally, in PCa cells, SUMO-PTM of PTEN promotes trafficking of PTEN outside the nucleus, specifically to the plasma membrane [14]. This is different from other cancer epithelial cell models in which PTEN SUMOylation can initiate nuclear recruitment [15]; hence the function of PTEN SUMOylation remains unclear. Currently, it is unknown whether SUMO-dependent PTEN localization works concentrically with or antagonizes ubiquitin-mediated PTEN degradation.

In the current manuscript, we demonstrate that induction of SENP1 protects PTEN from ubiquitin-dependent proteasomal degradation. Inversely, hyper-SUMO conditions support 1) SUMOylation of PTEN and WWP2, 2) PTEN nuclear exclusion, 3) greater PTEN-WWP2 interaction, and 4) PTEN ubiquitin-mediated proteolysis. The elevated PTEN serves as the primary PCa barrier in SENP1-Tg mice as reduction of PTEN facilitates micro-invasive cancers in SENP1-Tg mice. Hence, SENP1 inhibitors could serve as effective therapy for more aggressive PTEN-deficient prostate cancer.

## RESULTS

### Elevated SENP1 modulates cell apoptosis and PTEN protein stability in the prostate epithelia

**Figure 1:**
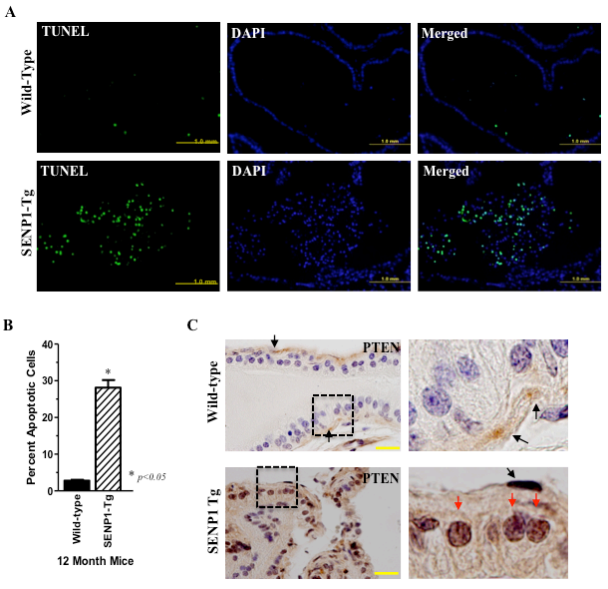
SENP1-Tg mice exhibit elevated prostate epithelial cells apoptosis and PTEN expression **A**. Tissue samples of the dorsolateral lobe of 12-month old SENP1-Tg and wild-type were independently immunodetected with TUNEL or DAPI-stained. **B**.Total DAPI- and TUNEL-positive nuclei were counted in the 40x magnification fields. The y-axis represents the percent of TUNEL-positive apoptotic cells to DAPI-stained cells in samples from SENP1-Tg (*n* = 7) *versus* wild-type (*n* = 3) mice; asterisk indicates statistical significance (*p* < 0.05) with Student's *t*-test analysis. **C**. Paraffin-embedded prostate tissue samples from wild-type and SENP1-Tg mice were exposed to the PTEN antibody and counter-stained with hematoxylin. Dashed box identifies the magnified region presented in the left panel, yellow scale bar represents 20 μm, and PTEN in basal cells (black arrows) and luminal epithelial nuclei (red arrows) is also indicated.

A large number of TUNEL-positive epithelial cells collect in the lumen of PG samples from 12-month old SENP1-Tg mice but are rarely observed in wild-type (Figure 1A) mice. However, SENP1-Tg mice also exhibit elevated prostate cell proliferation as previously reported [1]. Hence, to ensure that enhanced proliferation does not contribute to the difference between wild-type and SENP1-Tg mice, we normalized the number of TUNEL-positive cells to DAPI-stained nuclei in randomly selected 40x magnification field. Enhanced accumulation of apoptotic prostate epithelial cells is observed in samples from multiple 12-month old SENP1-Tg mice (*n* = 7) but clearly absent in their age-matched counterparts (*n* = 3, Figure 1B). Microarray analysis of prostate tissue isolated from 12-month old SENP1-Tg *versus* wild-type mice suggested a significant induction of pro-apoptotic genes associated with the canonical AKT/PTEN pathways in the SENP1-Tg mice (Supplementary Figure S1A and S1B). Since PTEN is a mediator of cellular apoptosis and a target for SUMO-modification, we evaluated the expression of PTEN protein in the prostate of SENP1-Tg mice. Wild-type mice exhibit positive PTEN expression in stem-like basal cells located on the PG periphery (black arrows, Figure 1C) but not luminal secretory epithelial cells. In contrast, prostate samples isolated from SENP1-Tg mice have a marked increase in PTEN expression in the luminal prostate epithelia (red arrows, Figure 1C). The elevated PTEN levels appear predominantly in the nuclei of the dysplasic prostate cells suggesting that SENP1 may regulate PTEN stability and nuclear retention.

### SENP1 regulates PTEN SUMOylation and ubiquitylation

**Figure 2:**
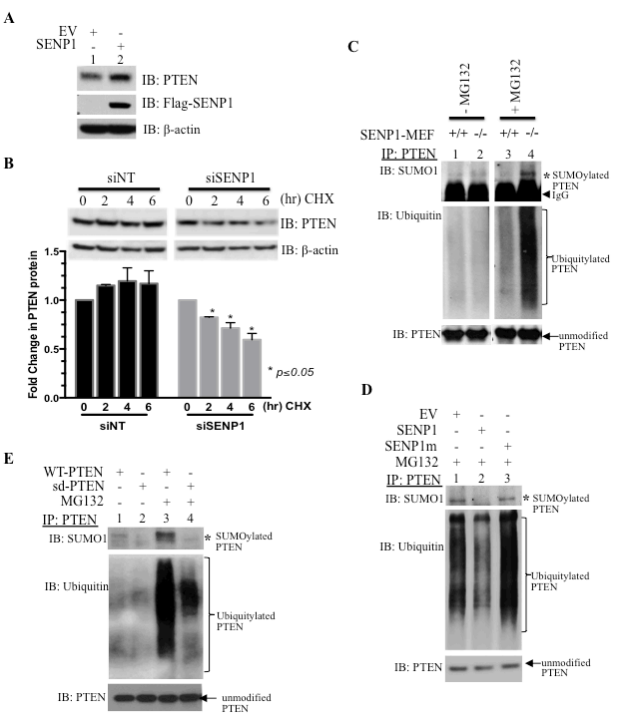
SENP1 regulates PTEN ubiquitylation to enhance protein stability **A**. Endogenous PTEN protein levels assessed in whole cell lysates (WCL) from HEK293 cells transfected with either empty vector (EV) or SENP1 using the indicated antibodies (IB); image is representative of 2 independent experiments. **B**. Cyclohexamide (100 μg/mL) was given for the indicated intervals to HEK293 cells pre-treated with non-targeting and SENP1-specific siRNA (siNT and siSENP1, respectively). After Western blotting, densitometry of PTEN and loading control β-actin was evaluated using ImageJ. The graph represents mean±SEM of the PTEN to β-actin ratio from two independent experiments; Student's *t*-test indicated statistical significance of *p*<0.05. **C**.-**D**. After the indicated treatments, cells were incubated for IP 16hr in the presence or absence of MG132 (10 μM). Endogenous PTEN was immunoprecipitated (IP) and subject to western blot analysis. Asterisk highlights SUMO1-modified PTEN while the bracket indicates the poly-ubiquitylated PTEN form. **C**. MEF cells were harvested from wild-type (+/+) or SENP1 knockout (−/−) mouse embryos. **D**. HEK293 cells were treated with plasmids for empty vector (EV), SENP1 or the inactive SENP1 mutant (SENP1m). **E**. PC3 cells were transfected with either wild-type or SUMO-deficient PTEN (WT and sd, respectively).

To examine this further, we sought to recapitulate a similar mechanism in cultured cells. Overexpression of SENP1 elevates endogenous PTEN levels in HEK293 whole cell lysates (Figure 2A). Cyclohexamide pulse-chase experiments were conducted with cells treated with SENP1-targeting siRNA (siSENP1) that produced a greater than 70% reduction in endogenous SENP1 mRNA as compared to non-targeting interference RNA (siNT, Supplementary Figure S2). Downregulation of endogenous SENP1 leads to a corresponding loss of PTEN protein (Figure 2B) indicating that SENP1 regulates PTEN protein stability.

Since PTEN is a known substrate for both SUMO and ubiquitin conjugation, we evaluated how SENP1 affects both these posttranslational modifications. First, we ensured that PTEN degradation is mediated *via* the proteasome; incubation with a proteasome inhibitor MG132 (10 μM) successfully inhibits PTEN loss (Supplementary Figure S3A). In the absence of MG132, neither SUMO nor ubiquitin PTM can be detected on immunoprecipitated endogenous PTEN in either wild or SENP1-MEF cells (lanes 1-2, Figure 2C). However, endogenous SUMO1 conjugation to PTEN is readily observed with MG132 treatment (lane 4, Figure 2C). Enhanced conjugation of SUMO1 to PTEN was accompanied by poly-ubiquitylation of PTEN (lane 4, Figure 2C). Although PTEN is conjugated with SUMO2 poly-chains under *in vitro* conditions (arrows, Supplementary Figure S3B), endogenous modification of PTEN with either SUMO2 or SUMO3 (collectively, SUMO2/3) was not observed; this is consistent with a recent study that also reports more efficient SUMO1 conjugation than SUMO2 of PTEN [19].

To assess whether the simultaneous SUMO1 and ubiquitin modification of PTEN is regulated *via* the catalytic activity of SENP1, cells were transfected with either the active SENP1 or the inactive SENP1 mutant (SENP1m). Only wild-type SENP1, but not SENP1 mutant, is able to alter the conjugation of either SUMO1 or ubiquitin to PTEN (lane 3 *versus* lane 2, Figure 2D); therefore, SENP1's enzymatic activity causes the loss of both PTEN PTMs.

Since SENP1 is a SUMO-specific protease and does not exhibit de-ubiquitylating activity, it is possible that PTEN SUMOylation precedes the ubiquitin conjugation of PTEN. As identified previously [14], mutation of 2 SUMO-accepting lysine residues K254 and K266 to alanine generates a PTEN mutant (sd-PTEN) that cannot be conjugated by SUMO1 (lane 2, Figure 2E). MG132 treatment of PC3 PCa cells enhanced both the SUMOylated and ubiquitylated forms of PTEN (lane 1 *versus* lane 3, Figure 2E). In contrast, both modifications are dramatically reduced on the sd-PTEN as compared to the PTEN wild-type (lane 3 *versus* lane 4, Figure 2E). Therefore, SUMO-modification precedes ubiquitylation of PTEN. Consistently, the sd-PTEN mutant is more stable than wild-type PTEN as evaluated in whole cell lysates (Supplementary Figure S3C).

### SENP1 directs PTEN's subcellular localization and interaction with ubiquitin E3 ligase WWP2

**Figure 3:**
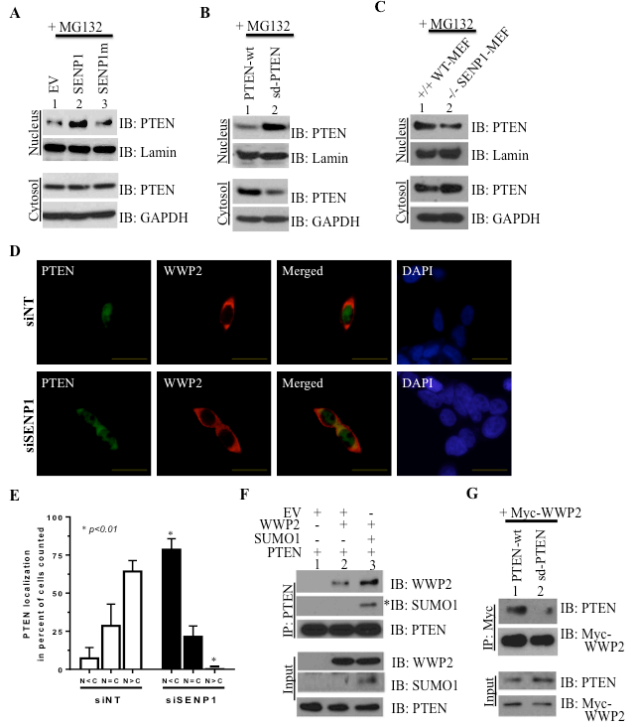
PTEN's subcellular localization and association with the ubiquitin ligase WWP2 is directed by SENP1. HEK293 **A**., LNCaP **B**., and MEF **C**. cells were treated as indicated and incubated with 10 μM MG132 16hr prior to isolation. Cell fractionation was performed and lysates were separated on SDS-PAGE to evaluate for protein levels of PTEN *versus* two markers lamin and GAPDH. All images represent 2-3 independent experiments. **D**.-**E**. PC3 cells were treated with SENP1- and non-targeting siRNA (siSENP1 and siNT, respectively) for 24 hr and subsequently transfected with PTEN and WWP2, simultaneously. After 8 hr, the cells were exposed to MG132 (10 μM) for 16 hr and immunofluorescence was performed with select antibodies for the indicated proteins while DAPI was used to identify cell nuclei. Representative cells at 60x magnification are shown with the scale bar at 25 μm. **E**. In 3 independent experiments, protein distribution of PTEN was assessed based on the following scenarios: 1) nuclear greater than cytosol (N > C), 2) nuclear equal to cytosol (N = C), or 3) nuclear less than cytosol (N < C). The percent of the total cell population that exhibited the specific condition was calculated and Student's *t*-test was utilized to evaluate statistical significance (asterisk indicates a p-value *≤0.01*). **F**. LNCaP cells were transfected with EV, PTEN, SUMO1, and WWP2 and subject to PTEN immunoprecipitation (IP) to identify SUMOylated PTEN (asterisk) and evaluate interaction with WWP2. **G**. LNCaP cells were transfected with Myc-tagged WWP2, the wild-type PTEN (PTEN wt), and SUMO-deficient PTEN (sd PTEN) and subsequently samples were immunoprecipitated with the anti-Myc antibody. Western blot analysis was conducted to evaluate complex formation between the Myc-WWP2 and either PTEN protein in 2 independent experiments.

Since nuclear retention can prevent the ubiquitin-dependent degradation of PTEN [20], we postulated that elevated SENP1 may direct PTEN subcellular localization in PCa cells. In LNCaP cells that lack endogenous PTEN, overexpressed PTEN is located outside the nucleus but stable SENP1 over-expression in these cells causes nuclear PTEN enrichment (Supplementary Figure S4A). This observation closely resembles the PTEN distribution in SENP1-Tg mice (Figure 1C). Consistently, HEK293 cell fraction reveals enhanced nuclear accumulation of PTEN with induction of wild-type, but not catalytically inactive, SENP1 (lane 2 *versus* lane 3, Figure 3A). The hypoSUMOylated sd-PTEN mutants are also enriched in nuclear fractions of LNCaP cells (lane 2, Figure 3B). Inversely, hyperSUMOylated PTEN is localized predominantly in the cytosol as observed in SENP1-null MEF (Figure 3C) and siSENP1-treated HEK293 cells (Figure 3D-3E). Collectively, the results suggest that SUMO PTM can direct the cellular distribution of PTEN with elevated SENP1 levels supporting PTEN nuclear retention.

We next evaluated how change in subcellular localization of PTEN affects its interaction with its major ubiquitin E3 ligase WWP2. WWP2 is located predominantly in the cytosol (control siNT-treated cells, Figure 3D and Supplementary Figure S4B). While SENP1-targeting siRNA does not alter WWP2 subcellular location, the hyper-SUMO condition does increase co-localization of WWP2 and PTEN due to the re-distribution of PTEN (siSENP1-treated cells, Figure 3D and Supplementary Figure S4B). Consistently in SENP1-deficient MEF cells, endogenous PTEN and WWP2 interact more readily (Figure S4C). Also, overexpression of SUMO1 potentiates both PTEN's SUMOylation and interaction with WWP2 as compared to native conditions (lane 2 *versus* lane 3, Figure 3F). The SUMO-deficient PTEN mutant that is retained in the nucleus (Figure 3B) does not associate with WWP2 (Figure 3G). Hence, SUMO-dependent translocation of PTEN to the cytosol facilitates association with WWP2.

**Figure 4:**
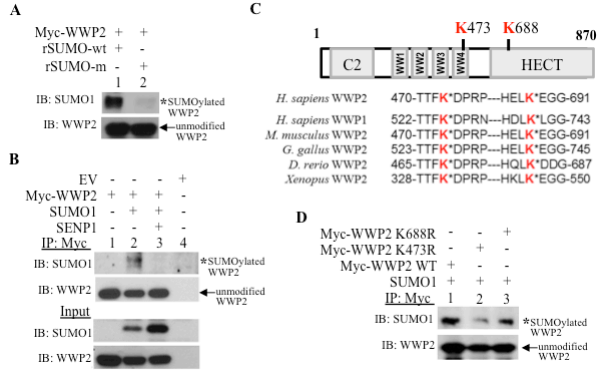
WWP2 is a substrate for SUMO modification **A**. Isolated Myc-tagged WWP2 was incubated with either wild-type or inactive-mutant recombinant SUMO1 (rSUMO-wt and rSUMO-m, respectively) in *in vitro* SUMOylation conditions. **B**. HEK293 cells were transfected with EV, Myc-tagged WWP2, SUMO1, and SENP1; harvested cells were subjected to immunoprecipitation (IP) with the Myc antibody and evaluated for the SUMO1-conjugated form of WWP2 (asterisks) *via* Western Blots. **C**. *In silico* analysis indicated two high-scoring potential SUMO-acceptor sites; K473 resides in the fourth WW-domain and K688 in the HECT catalytic domain are highlighted in red. Sequence alignment with an additional WWP isoform WWP1 and multiple WWP2 orthologs was conducted to evaluate for conservation of SUMO-targeted lysines. **D**. SUMO1-modification of wild-type WWP2, K473R, and K688R mutant was assessed in PC3 cells.

### SUMOylation of WWP2 increases association with PTEN

WWP2 is SUMO1-modified; in *in vitro* SUMOylation assays, incubation of isolated Myc-WWP2 with active, but not inactive, SUMO1 recombinant protein generates higher molecular weight WWP2 conjugates that are detectable with the SUMO1 antibody (Figure 4A). In cells, the WWP2 SUMO-modification band (lane 2, Figure 4B) is lost with induction of SENP1 (lane 3, Figure 4B). Hence SUMOylated WWP2, like PTEN, is a substrate for SENP1 isopeptidase activity. Using two SUMO prediction databases GPS-SUMO and SUMOplot, we identified two potential SUMO-acceptors sites on WWP2 that are conserved in a WWP isoform WWP1 and several orthologs (Figure 4C). The lysine residue K473 is located in the WW-domain while K688 resides in the HECT-catalytic region (Figure 4C). Site directed mutagenesis of K473 reduces WWP2 SUMOylation as compared to either K688 mutation or WWP2 wild-type (lane 2 *versus* either lane 1 or 3, Figure 4D). Therefore, K473 is the major SUMO-modification site of WWP2. The SUMO-acceptor K473 is critical for WWP2 function; K473R does not reduce PTEN levels (lane 4, Supplementary Figure S5A) or enhance global ubiquitylation (lane 4 Supplementary Figure S5B) as compared to wild-type WWP2 (lane 3, Supplementary Figure S5A and S5B, respectively).

**Figure 5:**
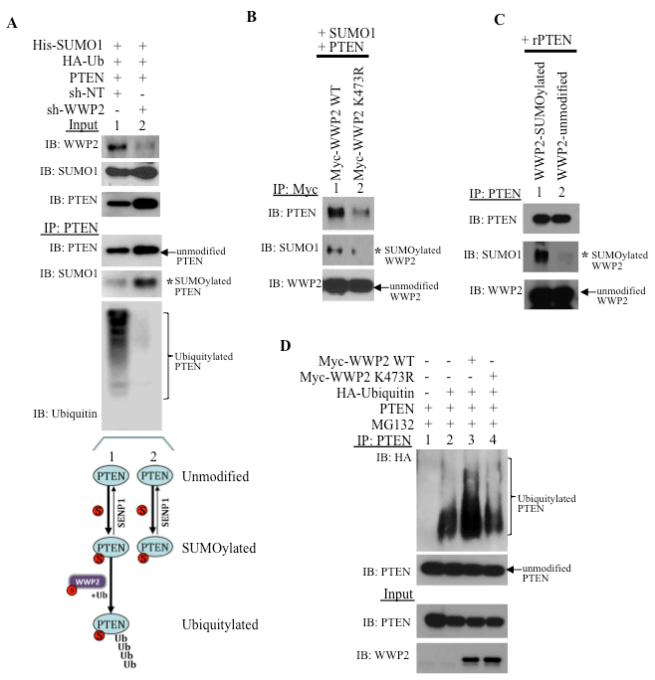
SUMOylation of WWP2 is critical for binding and ubiquitylating PTEN **A**. PC3 cells were incubated with non-targeting or WWP2-specific shRNA for 24 hr and subsequently transfected with His-SUMO1, HA-ubiquitin, and PTEN. After 8 hr, the cells were treated with 10 μM MG132 for an additional 16 hrs before isolation of PTEN protein. The schematic illustration demonstrates the multiple posttranslational modified forms of PTEN that persist. **B**. WWP2 wild-type and K473R mutant (Myc-WWP2 wt and WWP2-K473R, respectively) were transfected in HEK293 cells and subsequently isolated with the Myc antibody. Immunoblots demonstrating SUMO1-modification and PTEN interaction for isolated WWP2 constructs is shown. **C**. *In vitro* interaction between recombinant PTEN and SUMO1-modified and unmodified WWP2. **D**. PTEN protein was isolated from PC3 cells previously transfected with Myc-tagged wild-type or K473R WWP2.

In PC3 cells, WWP2-targeting shRNA (shWWP2) treatment significantly reduces endogenous WWP2 as compared to control non-targeting shRNA (shNT, Input, Figure 5A). Whereas PTEN is SUMOylated and ubiquitylated in shNT-treated cells (IP, lane 1, Figure 5A), SUMOylation but not ubiquitylation of PTEN is observed following shWWP2-treatment (IP, lane 2 Figure 5A). Hence, WWP2 is required for the subsequent ubiquitylation of SUMO-conjugated PTEN. SUMOylation of WWP2 facilitates association with PTEN as the SUMO-deficient WWP2 mutant K473R interacts poorly with PTEN as compared with its wild-type counterpart (Figure 5B). GPS-SUMO-based sequence analysis revealed two potential SUMO-interaction motifs (SIM) in the C2 and Phosphatase domains of PTEN (Supplementary Figure S6A). PTEN forms a non-covalent bond with SUMO1 and increasing amounts of a SIM peptide reduces the PTEN-SUMO1 interaction (Supplementary Figure S6B). Consistently, recombinant PTEN efficiently binds SUMO1-modified WWP2 in *in vitro* studies (Figure 5C). Loss of WWP2 SUMOylation retards PTEN ubiquitylation (Figure 5D). Pull-down of PTEN shows enhancement of PTEN ubiquitylation with expression of WWP2 wild-type (lane 3 *versus* lane 2, Figure 5D) but, the WWP2-K473R mutant does not enhance PTEN ubiquitin-PTM (lane 4, Figure 5D). In contrast, the minor SUMO1 site mutant K688R does bind and prompt poly-ubiquitylation of PTEN (Supplementary Figure S7A and S7B).

### Reduction of PTEN required for prostate cancer formation in SENP1-Tg mice

**Figure 6:**
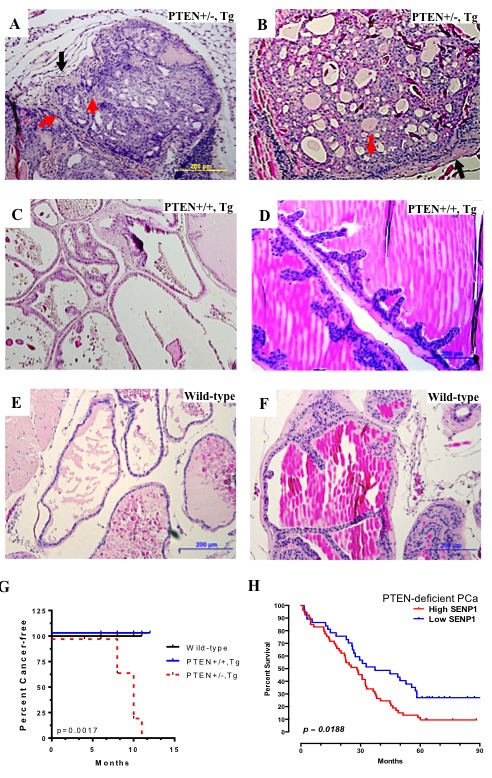
PTEN reduction induces carcinogenesis in SENP1 Transgenic mice From 10 month old PTEN+/−, Tg (**A**-**B**), PTEN+/+, Tg (**C**-**D**), and wild-type (**E**-**F**), the dorsolateral (A, C, E) and anterior (B,D,F) prostate lobes were harvested, paraffin-embedded, H&E stained, and assessed for histological changes. **G**. Assessment of additional mice and statistical analysis with Kaplan-Meier survival curves indicated that PTEN+/−, Tg (*n* = 9) were more susceptible to cancer than PTEN+/+,Tg (*n* = 9) or wild-type (*n* = 6) mice. **H**. Survival assessment of TCGA datasets of PCa patients with deletion of PTEN (*n* = 120) and high *versus* low SENP1 mRNA levels. Log-rank test was used to derive p-value.

Next, we postulated that absence of carcinoma in the SENP1-Tg mice was due primarily to the elevated PTEN levels. Hence, the SENP1-Tg mouse was crossed with a heterozygous PTEN mouse (PTEN+/−). We previously established that independently either SENP1 Tg or PTEN+/− mouse model develops PIN, but not prostate carcinoma [2, 21–23]. In contrast, the SENP1-Tg and PTEN+/−bigenic mouse (PTEN+/−,Tg) has local invasive carcinoma in the anterior and dorsolateral lobes of the prostate gland of 10-month mice (red arrows, Figure 6A and 6B) with corresponding thickening of the surrounding stroma (black arrow, Figure 6A and 6B). The aberrant morphology is more prominent in the anterior lobe of the PTEN+/−, Tg mice PG as loss of defined cell polarity, cribriform structures, and disruption of the stroma-gland barrier is noted (Figure 6B). Mice with normal PTEN and elevated SENP1 levels (PTEN+/+,Tg) exhibit low-grade PIN only in the dorsolateral lobe (Figure 6C) and no detectable dysplasic growth in the anterior lobe (Figure 6D). Age-matched wild-type mice exhibit normal prostate lobes (dorsolateral and anterior, Figure 6E and 6F, respectively). Assessment of additional mice supports that while wild-type and PTEN+/+,Tg survive cancer-free, PTEN+/−,Tg mice have a higher incidence of PCa (Figure 6G). Consistently, the survival rate of PTEN-deficient PCa patients is significantly lower with concurrent elevation of SENP1 mRNA (Figure 6H). Therefore, prostate carcinogenesis persists with both the loss of PTEN and induction of SENP1.

## DISCUSSION

The WW-domains of WWP2 recognize the consensus PPxY motif on cellular substrates to mediate the canonical mechanism for targeting the ubiquitin ligase activity of NEDD4 family members [24–27]. Although PTEN is a well-characterized substrate for WWP2 especially in PCa cells, the PTEN protein sequence lacks the established WW-recognition motif [18]. Therefore, it is unclear how PTEN is targeted for WWP2-mediated ubiquitylation. Interestingly, independent of covalent SUMO1-modification, PTEN also forms a non-covalent bound with SUMO1 (Supplementary Figure 6B). WWP2 is SUMOylated with the major SUMO1-acceptor site on the fourth WW-domain of WWP2 (Figure 4). The current results suggest that hyperSUMO conditions facilitate PTEN-WWP2 interaction. Specifically two regulatory mechanisms mediate this interaction: first, SUMOylation of PTEN supports protein subcellular redistribution and brings PTEN within closer proximity to WWP2 and second SUMOylation of WWP2 enhances binding of PTEN.

Previous studies have established that PTEN translocates to the nucleus *via* its nuclear localization signal-like sequence [28], diffusion through nuclear pores [29], and mono-ubiquitylation [21]. A recent study suggested a role for PTEN SUMOylation in subcellular distribution as SUMOylated PTEN in PCa cells is readily localized at the plasma membrane [14]; nuclear fractions were not investigated. Consistently, we now report that elevated SENP1 levels in PCa cells favors nuclear PTEN import (Figure 1C, 3A, and Supplementary Figure S4A). Our observations correlate with the previous study as both suggest that PTEN SUMOylation mediates the trafficking of PTEN to compartments outside the nucleus (Figure 3C, 3D, and 3E). Alternatively, our results differ from another report that demonstrates SUMO-mediated PTEN nuclear retention [15]. The contrast could be attributed to differences in 1) cell type as the Bassi and colleagues did not evaluate PCa cells and/or 2) the SUMO isoform conjugated to PTEN with SUMO1-PTEN in our system and SUMO2/3-PTEN in theirs. Additional reports in the literature support that SUMO1-modified PTEN could be excluded from the nucleus. First, Gonzalez-Santamaria and colleagues suggest that SUMO1-conjugation of PTEN decreases PTEN mono-ubiquitylation in an *in vitro* system [30]. Since PTEN mono-ubiquitylation allows the protein to translocate to the nucleus, it is possible that SUMO1 inhibits PTEN nuclear accumulation by antagonizing mono-ubiquitin-dependent nuclear import. Unfortunately, we were unable to detect endogenous mono-ubiquitylation of PTEN in our *in vivo* system. A second more likely scenario is associated with one of two critical SUMO-acceptor site (K266) that resides within a PTEN nuclear localization signal sequence (NLS, amino acids 265-269) [28]. The covalent binding of a SUMO1 moiety at K266 could hinder the recognition of the NLS for nuclear translocation; inversely, SENP1 can cleave the SUMO1 conjugated to PTEN (Figure 2D) and thereby support nuclear import.

Unlike the extensive literature on RING-ubiquitin ligases, little is known about the consequence of SUMO-PTM on the function of HECT-domain containing ubiquitin E3 ligases. This report demonstrates that SUMOylation of WWP2 directs substrate recognition. Specifically, 1) loss of the WWP2 SUMO1-acceptor site reduces WWP2-PTEN association (Figure 5B) and 2) SUMOylated *versus* unmodified WWP2 exhibits greater binding to PTEN (Figure 5C, and Supplementary Figure S7A). Many additional WWP2-substrates are well-established oncogenes (Oct4 and Sox9) and pro-metastatic factors (Smad3) in PCa [31–33]. Hence, an interesting area for future investigation would be to evaluate whether the elevated SENP1 modulates WWP2 ubiquitin E3 activity against these PCa regulators.

**Figure 7:**
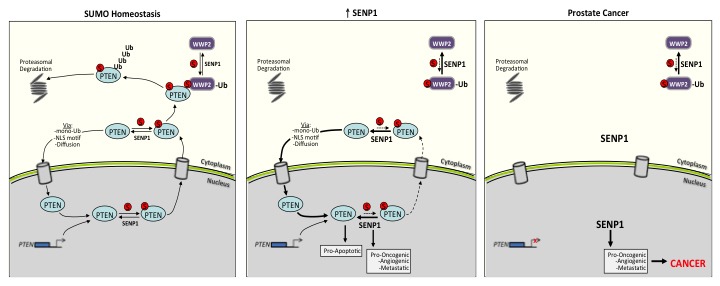
Schematic of the SENP1-regulated PTEN expression under normal SUMO homeostatic conditions *versus* prostate cancer onset The illustration is discussed in the manuscript.

Collectively, results from the present study further demonstrate how critical SUMO homeostasis is for normal cell biology. As illustrated in Figure 7, under normal physiological conditions, equilibrium is maintained between the level of SUMO-conjugated and unconjugated PTEN; the same is true for WWP2. This SUMO balance dictates PTEN's 1) normal distribution in the nucleus and cytosol and 2) poly-ubiquitin-mediated proteasomal degradation. With induction of SENP1, both SENP1-targets PTEN and WWP2 are sustained in the hypo-SUMOylated form. This significantly reduces PTEN-WWP2 interaction and subsequently PTEN degradation. In addition, the deSUMOylated PTEN more efficiently translocates to the nucleus to mediate multiple pro-apoptotic pathways [10]. Independently, elevated SENP1 initiates multiple pro-oncogenic, angiogenic, and metastatic mechanism delineated in our previous publications [1–3, 5]; the initiation of these pathways is sufficient to prompt dysplasic transformation in the mouse PG [2]. The progression of glandular cells from dysplasia to carcinoma requires a decrease in the population's rate of death [34]. Consistently, mutation to the pro-apoptotic gene *PTEN* is often observed with human prostate carcinogenesis [35]. Loss of PTEN expression allows for the elevated SENP1 to drive microinvasive carcinoma (Figure 6).

Multiple studies indicate that SENP1 induction directly correlates with prostate cancer severity and recurrence [5, 6]. SENP1 expression is regulated dependent and independent of androgen [6, 36] and elevated SENP1 levels, in turn, potentiate both androgen-dependent and independent PCa cell growth [5, 6]. Androgen-activated AR can directly bind to the *SENP1* promoter and potentiate gene transcription [6]; this initiates a positive feedback loop in which the elevated SENP1 increases AR-dependent cell growth *via* deSUMOylation of the co-regulator HDAC1 [37]. SENP1 also exhibits an analogous self-enforcing loop with HIFα to facilitate androgen-independent PCa cell proliferation [2, 5, 7]. Taken collectively with the current results, these findings provide potential mechanistic insight into development of castration-resistant PCa. Anti-androgen therapy (ADT) in advanced PCa would initially benefit patients with high PTEN expression as androgen-dependent SENP1 levels would be reduced. However, the SENP1 loss causes SUMO/ubiquitin-mediated PTEN degradation (Figure 2) and thereby could exaggerate PI3K signal transduction [14]. PI3K promotes HIF1a expression [38], which would restore SENP1 levels and support androgen-independent cell growth.

In advanced PCa patients with low PTEN and/or mutated *PTEN*, ADT would be slightly more effective. These PCa cells would be acclimated to low-PTEN/ high-PI3K signaling and therefore treatment with ADT would be less efficient at potentiating the PI3K pathway. Instead, ADT modestly decreases SENP1 levels and thereby reduces SENP1-regulated pro-oncogenic cascades [6]. However, in the same scenario, directly targeting SENP1 could prove as better anti-cancer therapy. Molecules that disturb SENP1's catalytic activity would successfully inhibit both androgen-dependent and -independent pro-oncogenic/metastatic signaling in low PTEN-expressing advanced PCa disease.

## MATERIALS AND METHODS

### Cell cultures, plasmids, and antibodies

LNCaP, PC3, HEK293, wild-type MEF, and SENP1-deficient MEF cells were maintained as previously published [6, 7]. The Flag-tagged SENP1 SENP1m, and PTEN vector plasmids were previously generated [39, 40]. The following antibodies and recombinant proteins were utilized: PTEN (A2B1; mouse-monoclonal from Santa Cruz and 138G6; rabbit-monoclonal from Cell Signaling, Beverly, MA, USA), SUMO1 (Cell Signaling), Ubiquitin (P4D1, Santa Cruz), Flag (M2; Sigma-Adrich, St Louis, MO, USA), Actin (Santa Cruz), Lamin (Santa Cruz), GAPDH (ab9484; abcam, Cambridge, MA, USA), and recombinant PTEN (MyBioSource, San Diego, CA, USA).

### Generation of SENP1 transgenic and PTEN heterozygous compound mice

Previously we generated and characterized the C-line SENP1-Tg mice that overexpressed the mouse SENP1 transgene in the prostate [2]. The well-characterized PTEN+/− mouse generated in Dr. Lin's laboratory [35] was bred with the C-line SENP1-Tg mice to produce a bigenic mouse model. Mouse tail-clips were utilized for genotyping; specifically PCR using the primers specific for the SENP1 transgene, GAPDH, [2] or PTEN (PTEN+/+: 5’-tgggaagaacctagcttggagg-3’; 5’-actctaccagcccaaggcccgg-3’ and PTEN+/−, 5’-ttccatttgtcacgtcctgcac-3’) was conducted. All mice were handled and used in accordance with the guidelines of the University of Texas MD Anderson Cancer Center and upon approval *via* the Institutional Animal Care and Use Committee.

### Mouse tissue isolation, histopathology, and immunohistochemistry

Mouse prostate tissue samples were processed and analyzed as previously described [2]. After micro-dissection, mouse prostate lobes were first examined for gross abnormalities including enlarged glands, tumor formation, and/or necrosis.

### Apoptosis assay

Apoptotic cells were identified using the TUNEL kit (*In Situ* Cell Death Detection Kit-Fluorescein, Roche, Branford, CT, USA) according to manufacturer's instructions. Subsequently, the total number of nuclei immunodetected *via* TUNEL and DAPI were independently counted in randomly selected field magnified at 40x.

### Interference RNA treatment

As previously outlined [22], cells were exposed to either non-targeting or SENP1-directed siRNA (Dharmacon, Lafayette, CO, USA) and SENP1 knockdown efficiency was confirmed using real-time PCR prior to use. For knockdown of WWP2, PC3 cells were infected with non-silencing control or WWP2-targeting shRNA lentiviral particles (ThermoScientific, Waltham, MA, USA) and harvested two days post transduction.

### Immunoprecipitation, SDS-Page, and immunoblotting

Harvested cells were subject to immunoprecipitation, lysates were resolved on SDS-PAGE, and western blot analysis was performed as previously described [41]

### *In vitro* WWP2 SUMOylation and PTEN interaction

WWP2 proteins were SUMO1 modified using SUMOlink (Active Motif, Carlsbad, CA, USA) according to the manufacturer's instructions. Briefly, recombinant WWP2 protein (Abnova) was incubated for 5 hr with SAE1/SAE2, Ubc9, and either SUMO1 or SUMO1-inactive mutant and then immunoprecipitated with the appropriate antibody. Subsequently the isolated WWP2 was incubated with recombinant PTEN protein (Cayman Chemical) for 16 hr at 4C. Interaction between PTEN and either SUMO-modified *versus* un-modified WWP2 was evaluated with SDS/PAGE and immunoblotting.

### Cell fractionation

The cytosolic and nuclear subcellular fractions were isolated with the kit (N-PER Nuclear and Cytoplasmic Extraction Reagents, Thermo-Scientific) using the provided protocol.

### Data analysis

The GraphPad Prism Version 4.0 (GraphPad Software) was used to evaluate for statistical significance *via* the Student's *t-test*. The same software was also employed to perform the log-rank test on Kaplan-Meier survival curves for the incidence of cancer in the wild-type *versus* bigenic mouse line.

Supplementary Information accompanies the paper on the Oncotarget website.

## SUPPLEMENTARY MATERIALS FIGURES


