# Supplementary material for: SENP1 regulates PTEN stability to dictate prostate cancer development
Source: Oncotarget. 2016 Nov 10;8(11):17651–64. doi: 10.18632/oncotarget.13283 (PMC5392276; doi:10.18632/oncotarget.13283)
Supplement: Supplementary file 1 [file oncotarget-08-17651-s001.pdf]

SENP1 regulates PTEN stability to dictate prostate cancer development

Supplementary Material

A

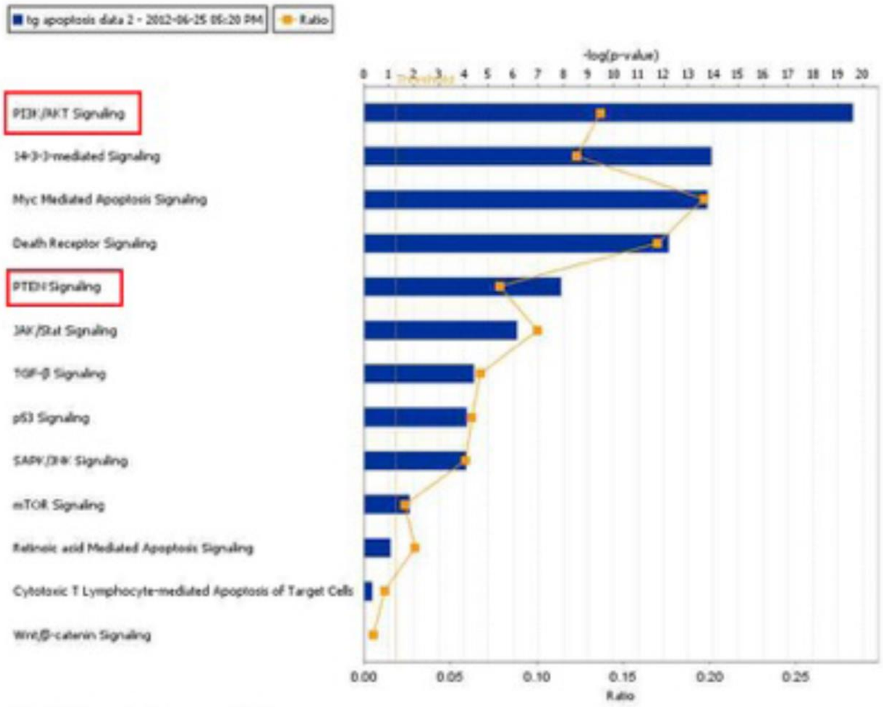

B

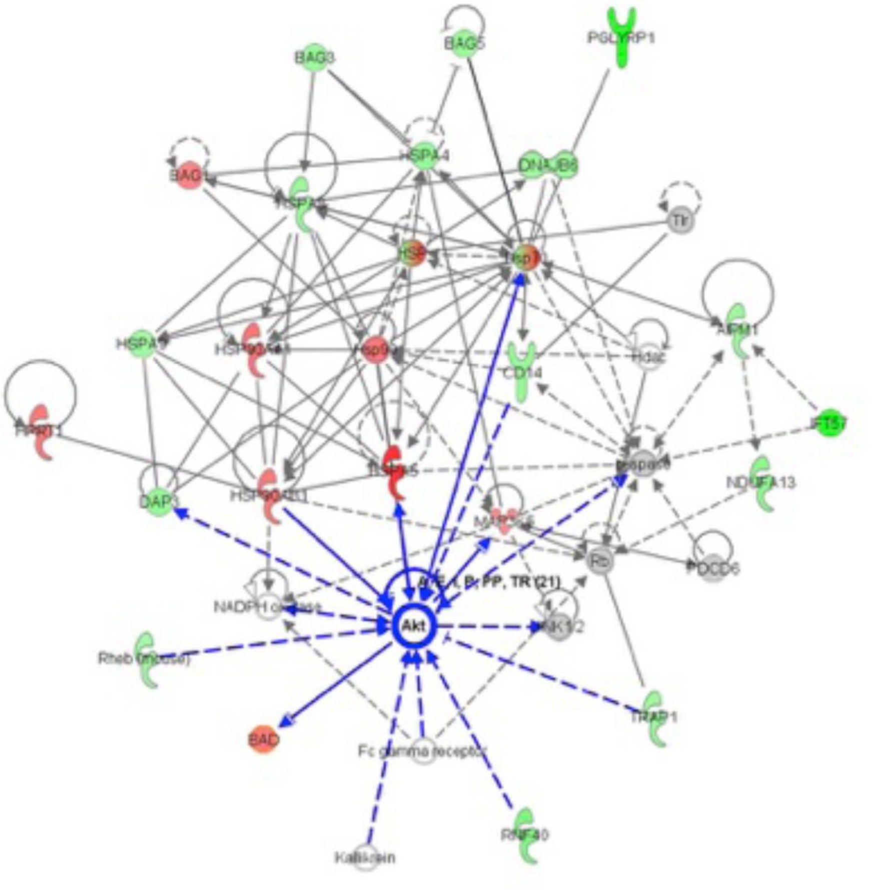

Supplementary Figure S1: SENP1 induction on canonical apoptotic pathways in the mouse PG.

The dorsolateral lobes from 12-month old SENP1 Tg and wild-type mice were micro-dissected,

pooled, subject to Microarray, and analyzed using the Ingenuity Software. (**A**) The analysis was performed to identify the canonical apoptotic pathway altered with induction of SENP1. (**B**) Specific components of the Akt pathway affected with SENP1 overexpression in the PG.

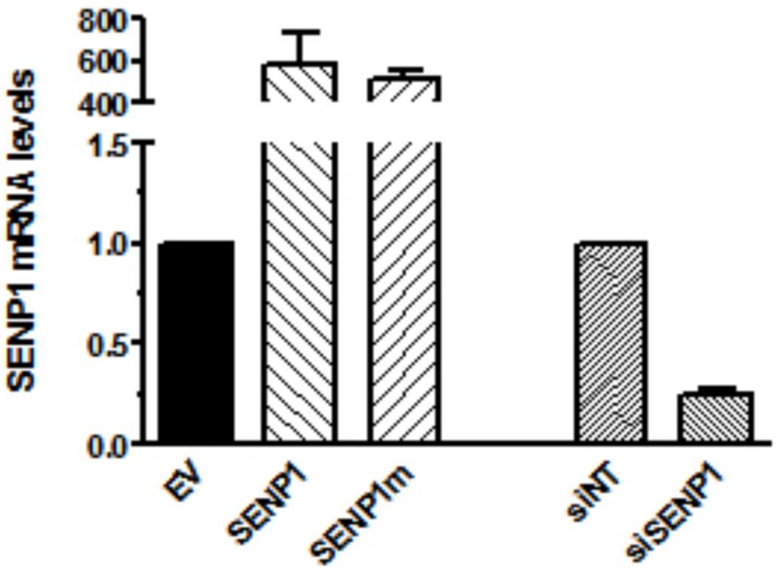

Supplementary Figure S2: SENP1 overexpression and knockdown efficiency and effects on PTEN subcellular localization. To evaluate SENP1 mRNA levels, real-time PCR analysis was performed after 48 hr transient SENP1 and SENP1m overexpression or various siRNA SENP1-targeted versus non-targeted treatment (siSENP1 and siNT respectively).

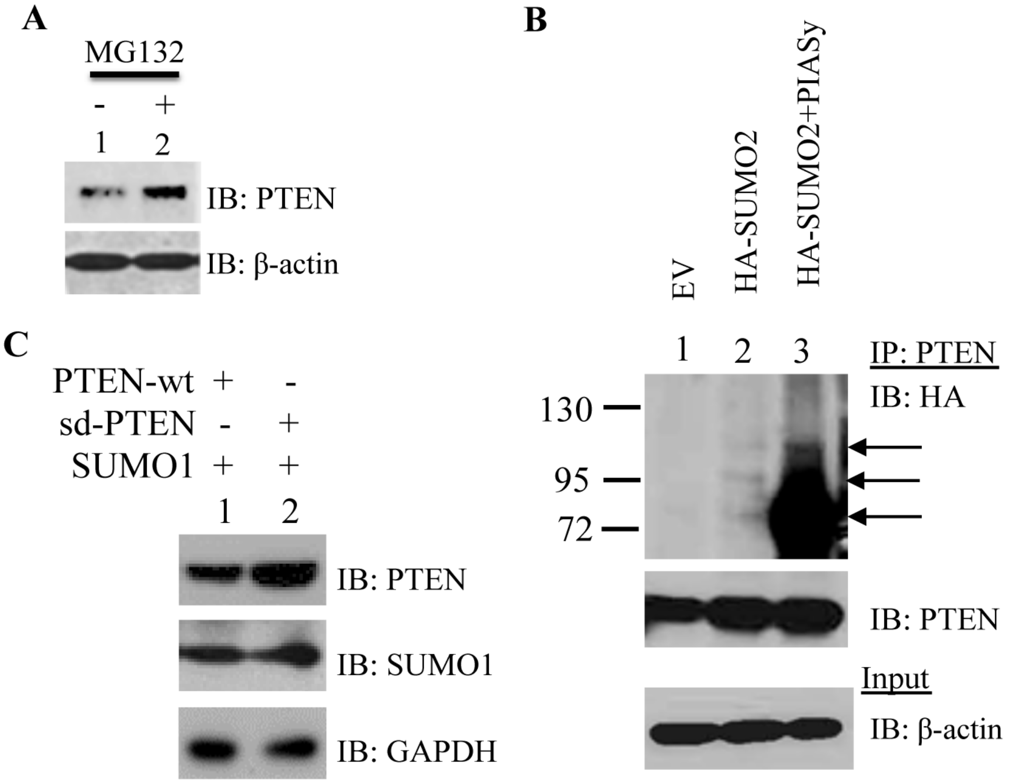

Supplementary Figure S3: In vitro PTEN SUMOylation and the stability of wild-type and SUMO-deficient PTEN. (**A**) The treatment of HEK293 cells with 10 μM MG132 for 16 hr enhances endogenous PTEN protein levels as compared to β-actin control. (**B**) SUMOylation assays indicate that endogenous PTEN can be conjugated to SUMO2 and SUMOylation can be potentiated with overexpression of the SUMO E3 ligase PIASy. (**C**) In LNCaP cells, the SUMO-deficient PTEN (sd PTEN) is more stable than PTEN wild-type (PTEN wt) after MG132.

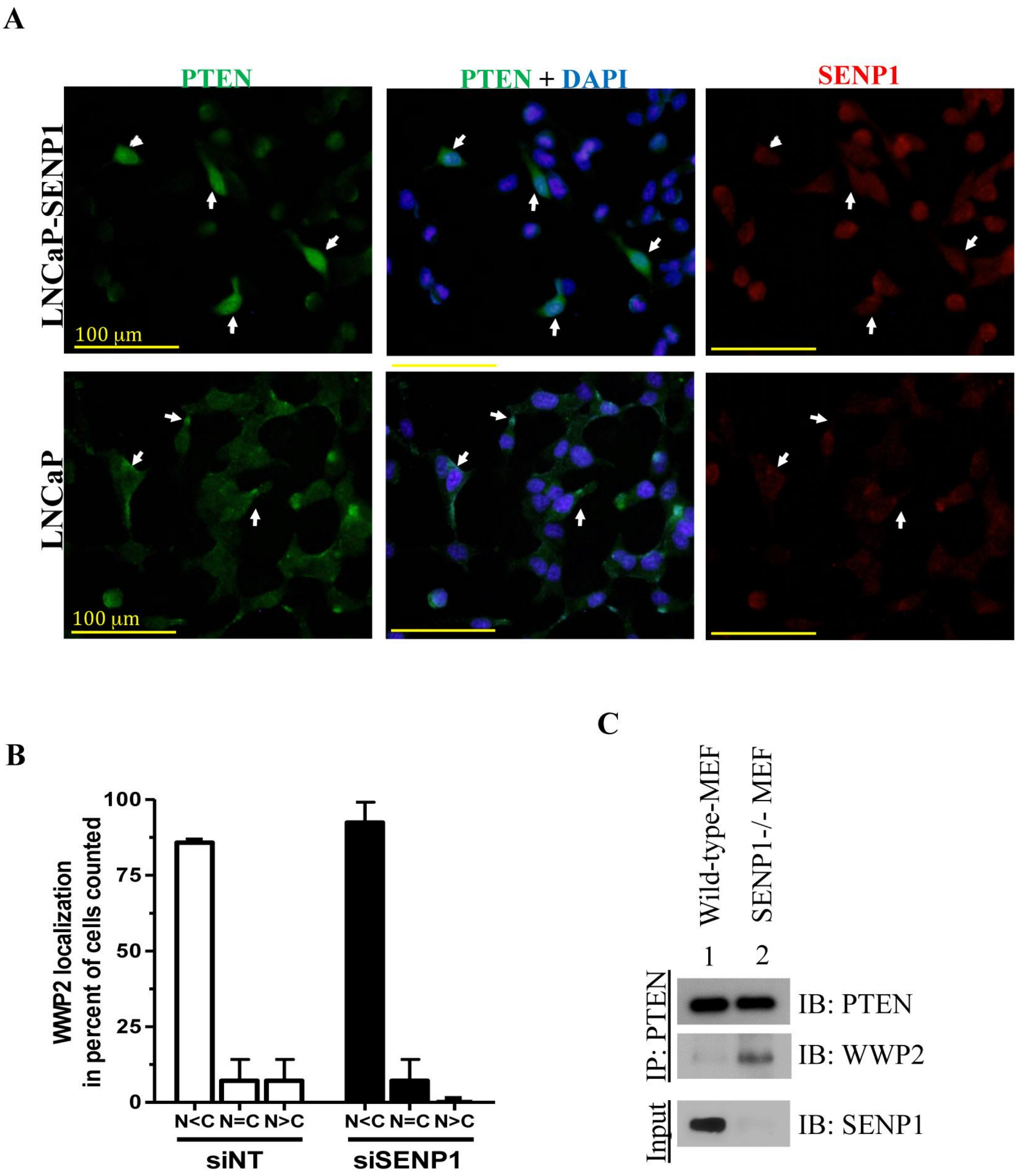

Supplementary Figure S4: SENP1 alters subcellular localization of PTEN, but not WWP2 while hyperSUMO conditions support PTEN-WWP2 interaction. (**A**) PTEN was expressed in parental LNCaP and LNCaP cells that stably overexpress SENP1. Immunofluorescence analysis reveals a considerable difference in the subcellular distribution of PTEN; SENP1 induction enhances nuclear compartmentalization of PTEN. (**B**) PC3 cells were treated with either non-targeting or SENP1-specific siRNA for 24 hrs and subsequently assessed for WWP2 distribution analogous

to PTEN in **Fig. 3D-E.** (**C**) Wild-type and SENP1-deficient MEF cells were harvested, immunoprecipitated for endogenous PTEN, and isolated fractions were evaluated for interaction with endogenous WWP2.

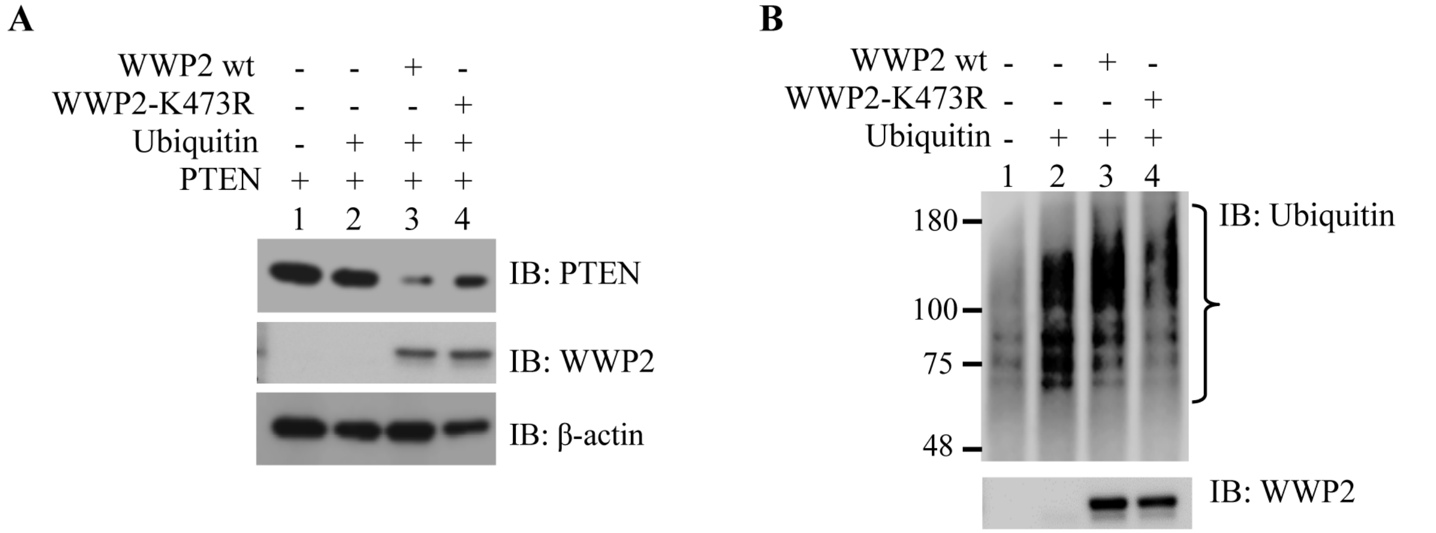

Supplementary Figure S5: WWP2 SUMOylation dictates PTEN protein stability and WWP2's Ubiquitin E3 ligase activity. PC3 cells were transfected with the indicated plasmids and treated with 10 μM MG132 for 16 hr. (**A**) PTEN is reduced with overexpression of the wild-type WWP2 (WWP2 wt) unlike the SUMO-deficient WWP2 (WWP2-K473R). (**B**) The high molecular weight poly-ubiquitin chain is decreased with overexpression of WWP2-K473R as compared to WWP2 wt.

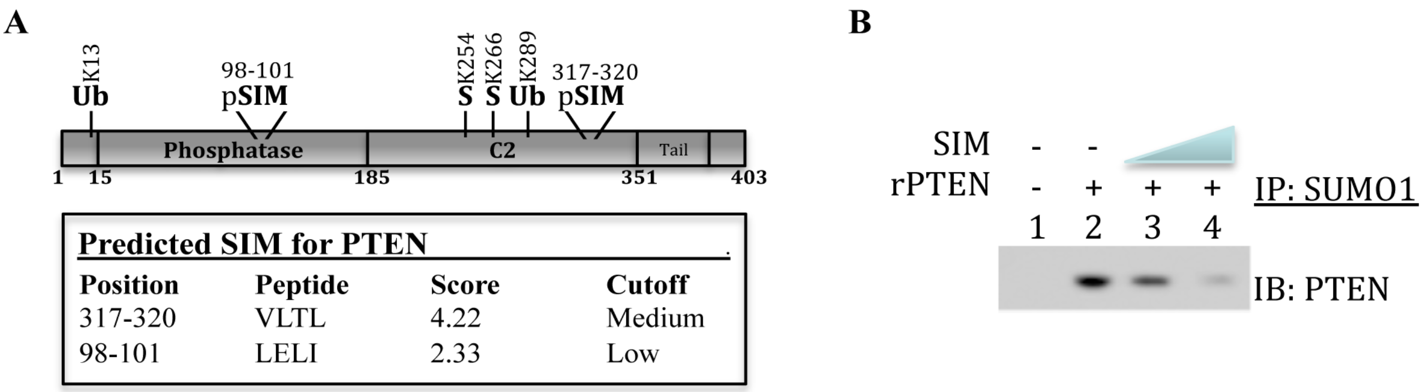

Supplementary Figure S6: PTEN exhibits non-covalent interaction with SUMO1. (**A**) Schematic illustrates location of known ubiquitin and SUMO-acceptor lysine residues and consensus SIMs (labeled pSIM) on PTEN amino acid sequence. The SIMs were identified using *in silico* GPS-SUMO assessment with the respective scores presented. (**B**) *In vitro* competitive binding between recombinant PTEN and increasing amounts of the SIM peptide (100 and 200 ng) for SUMO1.

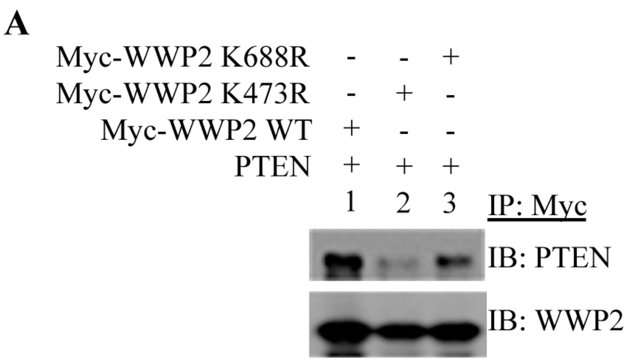

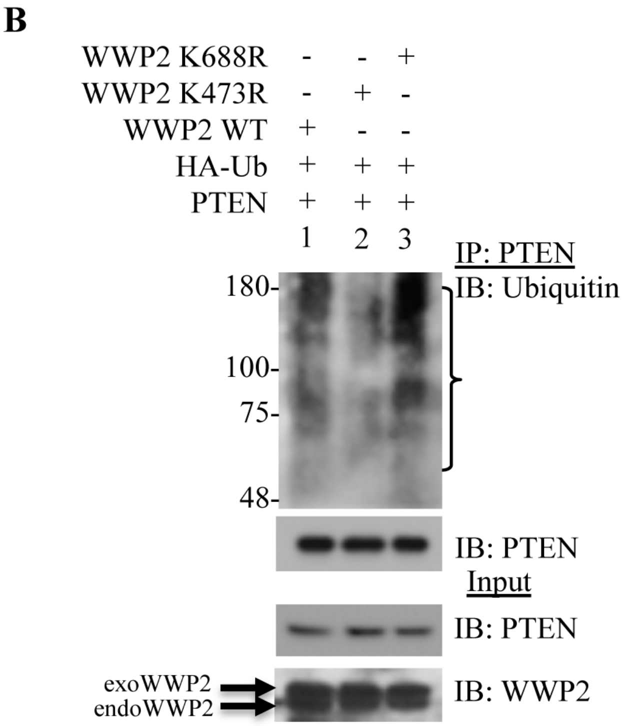

Supplementary Figure S7: WWP2 SUMOylation enhances association with and ubiquitylation of PTEN. HEK293 cells were transfected with the indicated plasmids and incubated with 10 μM MG132 16hr prior to isolation. Proteins were isolated with the either the Myc (**A**) or PTEN (**B**) antibody, and lysates were subject to SDS-PAGE Western Blot analysis.
